# Abstinence-Only Education and Teen Pregnancy Rates: Why We Need Comprehensive Sex Education in the U.S

**DOI:** 10.1371/journal.pone.0024658

**Published:** 2011-10-14

**Authors:** Kathrin F. Stanger-Hall, David W. Hall

**Affiliations:** 1 Department of Plant Biology, The University of Georgia, Athens, Georgia, United States of America; 2 Department of Genetics, The University of Georgia, Athens, Georgia, United States of America; Indiana University, United States of America

## Abstract

The United States ranks first among developed nations in rates of both teenage pregnancy and sexually transmitted diseases. In an effort to reduce these rates, the U.S. government has funded abstinence-only sex education programs for more than a decade. However, a public controversy remains over whether this investment has been successful and whether these programs should be continued. Using the most recent national data (2005) from all U.S. states with information on sex education laws or policies (N = 48), we show that increasing emphasis on abstinence education is positively correlated with teenage pregnancy and birth rates. This trend remains significant after accounting for socioeconomic status, teen educational attainment, ethnic composition of the teen population, and availability of Medicaid waivers for family planning services in each state. These data show clearly that abstinence-only education as a state policy is ineffective in preventing teenage pregnancy and may actually be contributing to the high teenage pregnancy rates in the U.S. In alignment with the new evidence-based Teen Pregnancy Prevention Initiative and the *Precaution Adoption Process Model* advocated by the National Institutes of Health, we propose the integration of comprehensive sex and STD education into the biology curriculum in middle and high school science classes and a parallel social studies curriculum that addresses risk-aversion behaviors and planning for the future.

## Introduction

The appropriate type of sex education that should be taught in U.S. public schools continues to be a major topic of debate, which is motivated by the high teen pregnancy and birth rates in the U.S., compared to other developed countries [1]–[4] (Table 1). Much of this debate has centered on whether abstinence-only versus comprehensive sex education should be taught in public schools. Some argue that sex education that covers safe sexual practices, such as condom use, sends a mixed message to students and promotes sexual activity. This view has been supported by the US government, which promotes abstinence-only initiatives through the Adolescent Family Life Act (AFLA), Community-Based Abstinence Education (CBAE) and Title V, Section 510 of the Personal Responsibility and Work Opportunity Reconciliation Act of 1996 (welfare reform), among others [5]. Funding for abstinence-only programs in 2006 and 2007 was $176 million annually (before matching state funds) [5], [6]. The central message of these programs is to delay sexual activity until marriage, and under the federal funding regulations most of these programs cannot include information about contraception or safer-sex practices [5], [7].

**Table 1:** U.S. teenage pregnancy and birth rates are high compared to other developed countries.

International Data	U.S.	France	Germany	Netherlands	Canada	UK
Pregnancy rate (2002–5)	72.2	25.7	18.8	11.8	29.2	41.3∧
Birth rate (2006)	41.9	7.8	10.1	3.8	13.3	26.7

Rates are listed as numbers per 1000 girls 15–19 years old,

∧ 15–18 years old [1]–[4].

The federal funding for abstinence-only education expired on June 30, 2009, and no funds were allocated for the FY 2010 budget. Instead, a “Labor-Health and Human Services, Education and Other Agencies” appropriations bill including a total of $114 million for a new evidence-based Teen Pregnancy Prevention Initiative for FY 2010 was signed into law in December 2009. This constitutes the first large-scale federal investment dedicated to preventing teen pregnancy through research- and evidence-based efforts. However, despite accumulating evidence that abstinence-only programs are ineffective [6], [8], abstinence-only funding (including Title V funding) was restored on September 29, 2009 [8] for 2010 and beyond by including $250 million of mandatory abstinence-only funding over 5 years as part of an amendment to the Senate Finance Committee's health-reform legislation (HR 3590, Amendment #2786, section 2954). This was authorized by the legislature on March 23, 2010 [9].

With two types of federal funding programs available, legislators of individual states now have the opportunity to decide which type of sex education (and which funding option) to choose for their state, while pursuing the ultimate goal of reducing teen pregnancy rates. This large-scale analysis aims to provide scientific evidence for this decision by evaluating the most recent data on the effectiveness of different sex education programs with regard to preventing teen pregnancy for the U.S. as a whole. We used the most recent teenage pregnancy, abortion and birth data from all U.S. states along with information on each state's prescribed sex education approach to ask “what is the quantitative evidence that abstinence-only education is effective in reducing U.S. teen pregnancy rates?” If abstinence education results in teenagers being abstinent, teenage pregnancy and birth rates should be lower in those states that emphasize abstinence more. Other factors may also influence teenage pregnancy and birth rates, including socio-economic status, education, cultural influences [10]–[12], and access to contraception through Medicaid waivers [13]–[15] and such effects must be parsed out statistically to examine the relationship between sex education and teen pregnancy and birth rates. It was the goal of this study to evaluate the current sex-education approach in the U.S., and to identify the most effective educational approach to reduce the high U.S. teen pregnancy rates. Based on a national analysis of all available state data, our results clearly show that abstinence-only education does not reduce and likely increases teen pregnancy rates. Comprehensive sex and/or STD education that includes abstinence as a desired behavior was correlated with the lowest teen pregnancy rates across states. In alignment with the *Precaution Adoption Process Model* advocated by the National Institutes of Health we suggest that comprehensive sex and HIV/STD education should be taught as part of the biology curriculum in middle and high school science classes, along with a social studies curriculum that addresses risk-aversion behaviors and planning for the future.

## Materials and Methods

### Level of emphasis on abstinence in state laws

Data on abstinence education were retrieved from the Education Commission of the States [16]. Of the 50 U.S. states, only 38 states had sex education laws (as of 2007; Table 2). Thirty of the 38 state laws contained abstinence education provisions, 8 states did not. Following the analysis of the Editorial Projects in Education Research Center [17], which categorizes the data on abstinence education into four levels (from least to most emphasis on abstinence: no provision, abstinence covered, abstinence promoted, abstinence stressed), we assigned ordinal values from 0 through 3 to each of these four categories respectively. A higher category value indicates more emphasis on abstinence with level 3 stressing abstinence only until marriage as the fundamental teaching standard (similar to the federal definition of abstinence-only education), if sex or HIV/STD education is taught (sex education is not required in most states) [16]–[18]. The primary emphasis of a level 2 provision is to promote abstinence in school-aged teens if sex education or HIV/STD education is taught, but discussion of contraception is not prohibited. Level 1 covers abstinence for school-aged teens as part of a comprehensive sex or HIV/STD education curriculum, which should include medically accurate information on contraception and protection from HIV/STDs [16]–[18]. Level 0 laws on sex education and/or HIV education do not specifically mention abstinence.

**Table 2:** Abstinence provisions and levels of abstinence education in state laws & policies.

State	Law: Abstinence^1^	Law Level^2^	Laws & Policy Level^3^
Alabama	Yes	3	3
Alaska	-	-	1
Arizona	Yes	2	3
Arkansas	Yes	2	3
California	Yes	1	1
Colorado	Yes	2	2
Connecticut	No	0	0
Delaware	-	-	3
Florida	Yes	3	3
Georgia	Yes	2	2
Hawaii	-	-	3
Idaho	No	0	0
Illinois	Yes	3	3
Indiana	Yes	3	3
Iowa	No	0	0
Kansas	-	-	0
Kentucky	-	-	3
Louisiana	Yes	3	3
Maine	Yes	1	1
Maryland	-	-	0
Massachusetts	No	*0*	*1*
Michigan	Yes	1	1
Minnesota	Yes	1	1
Mississippi	Yes	3	3
Missouri	Yes	2	2
Montana	-	-	0
Nebraska	-	-	2
Nevada	No	0	0
New Hampshire	No	0	0
New Jersey	Yes	1	1
New Mexico	-	-	3
New York	-	-	1
North Carolina	Yes	3	3
North Dakota	-	-	-
Ohio	Yes	3	3
Oklahoma	Yes	3	3
Oregon	Yes	1	1
Pennsylvania	Yes	*2*	*3*
Rhode Island	Yes	*2*	*3*
South Carolina	Yes	3	3
South Dakota	Yes	2	2
Tennessee	Yes	3	3
Texas	Yes	3	3
Utah	Yes	3	3
Vermont	Yes	1	1
Virginia	Yes	2	2
Washington	Yes	2	2
West Virginia	No	0	0
Wisconsin	No	*0*	*1*
Wyoming	-	-	-

1 State laws with (yes) or without (no) an abstinence provision as of 2007 [16].

2 Level of Abstinence provision in state law as of 2007 [17].

3 Level of Abstinence provision in state law or other policy as of 2005 [19]; differences to laws^2^ are noted in *italics*.

### Level of emphasis on abstinence in state laws & policies

States without sex education laws may nevertheless have policies regarding sex and/or HIV/STD education. These policies may be published as Health Education standards or Public Education codes [19]. These policies can also provide information on how existing sex education laws may be interpreted by local school boards. Information on the sex education laws and policies for all 50 US states was retrieved from the website of the Sexuality Information and Education Council of the US (SIECUS). We analyzed the 2005 state profiles on sex education laws and policy data for all 50 states [19] following the criteria of the Editorial Projects in Education Research Center [17] to identify the level of abstinence education (Table 2). The coding for the state laws (N = 38) and the coding for both laws and policies (N = 48) was more or less the same for the states represented in both data sets with 6 exceptions (Table 2): the additional information on policies moved two states from a level 0 (abstinence not mentioned) to level 1 (abstinence covered), and four states from a level 2 abstinence provision (abstinence emphasized) to a level 3 (abstinence stressed). Only two states had neither a state law nor a policy regarding sex or STD/HIV education (as of 2005): North Dakota and Wyoming. Analyses of the two data sets gave essentially identical results. In this paper we present the analyses of the more extensive (48 states) law and policy data set.

### Teen pregnancy, abortion and birth data

Data on teen pregnancy, birth and abortion rates were retrieved for the 48 states from the most recent national reports, which cover data through 2005 [11], [12]. The data are reported as number of teen pregnancies, teen births or teen abortions per one thousand female teens between 15 and 19 years of age. In general, teen pregnancy rates are calculated based on reported teen birth and abortion rates, along with an estimated miscarriage rate [12]. We used these data to determine whether there is a significant correlation between level of prescribed abstinence education and teen pregnancy and birth rates across states. The expectation is that higher levels of abstinence education will be correlated with higher levels of abstinence behavior and thus lower levels of teen pregnancy.

### Other factors

Data on four possibly confounding factors were included in our analyses.

#### Socio-economics

To account for cost-of-living differences across the US, we used the adjusted median household income for 2006 for each state from the Council for Community and Economic Research: C2ER [20]. These data are based on median household income from the *Current Population Survey for 2006* from the U.S. Census Bureau [21] and the 2006 cost of living index (COLI).

#### Educational attainment

As an estimate of statewide education levels among teens, we used the percentage of high school graduates that took the SAT in 2005/2006 in each state [22].

#### Ethnic composition

We determined the proportion of the three major ethnic groups (white, black, Hispanic) in the teen population (15–19 years old) for each state [12], and assessed whether the teen pregnancy, abortion and birth rates across states were correlated with the ethnic composition of the teen population. To account for the ethnic diversity among the teen populations in the different states in a multivariate analysis of teen pregnancy and birth rates, we included only the proportion of white and black teens in the state populations as covariates, because the Hispanic teen population numbers were not normally distributed (see below).

#### Medicaid waivers for family planning

Medicaid-funded access to contraceptives and family planning services has been shown to decrease the incidence of unplanned pregnancies, especially among low-income women and teens [13]. According to the Guttmacher Institute, the national family planning program prevents 1.94 million unintended pregnancies, including almost 400,000 teen pregnancies each year by providing millions of young and low-income women access to voluntary contraceptive services [13], Medicaid covered 71% of expenditures for these programs in 2006, and it is estimated that states saved $4 (associated with unintended births) for each $1 spend on contraceptive services [13]. Since the increasing role of Medicaid in funding family planning was mainly due to the efforts of 21 states to expand eligibility for family planning for low-income women who otherwise would not qualify for Medicaid, we analyzed whether these Medicaid waivers for family planning services (available in some states but not in others) could bias our results. We determined which states had received permission (as of 2005) from the Federal Medicaid program to extend Medicaid eligibility for family planning services to large numbers of individuals whose incomes are above the state-set levels for Medicaid enrollment [15]. We assessed whether the waivers (access to family planning services) had an effect on our analysis of teen pregnancy and birth rates across states, specifically whether they could bias our analysis with respect to the effects of the different levels of abstinence education.

### Statistical Analyses

#### Sample statistics

Using JMP 8 software [23], we tested all variables for normality (Goodness of Fit: Shapiro Wilkes Test; JMP 8.0). Except for teen abortion rates and Hispanic teen population data, all variables were normally distributed. The distribution of the Hispanic teen population across states was not normal: most states had relatively small Hispanic teen populations, and a few states had a relatively large population of Hispanic teens. Teen pregnancy and birth rate distributions included outliers, but these outliers did not cause the distributions within abstinence education levels to differ significantly from normal, thus all outliers were included in subsequent analyses. For all further statistical analyses we used SPSS [24].

#### Correlations

We used non-parametric (Spearman) correlations to assess relationships between variables, and for normally distributed variables we also used parametric (Pearson) correlations, but these results showed the same trends and significance levels as the non-parametric correlations. As a result, we only report the results for the non-parametric correlations here.

#### Multivariate analyses

Only the two normally distributed dependent variables were included in the multivariate analysis (MANOVA and MANCOVA [24]): teen pregnancy and teen birth rates. We tested for homogeneity of error variances (Levene's Test) and for equality of covariance matrices (Box test) between groups. For MANCOVA we report the estimated marginal means of teen pregnancy and birth rates (i.e. means after the influence of covariates was removed). For pairwise comparison between abstinence levels, we used the Bonferroni adjustment for multiple comparisons.

## Results

**Figure 1:**
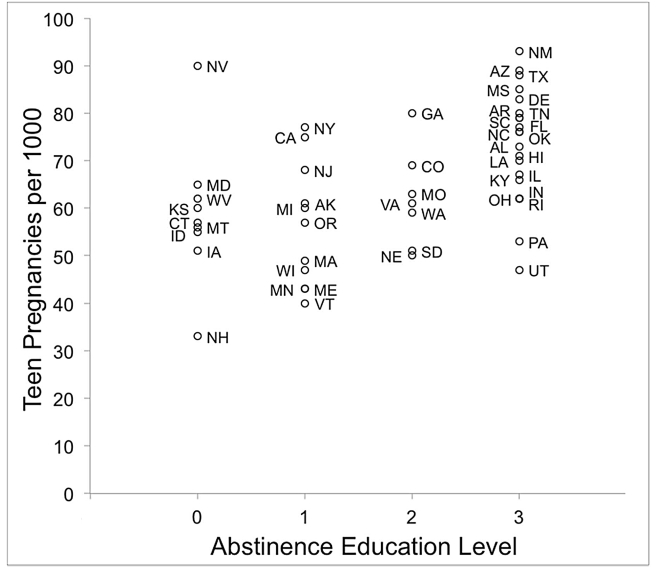
Abstinence education level prescribed in 2005 state laws or policies. All 48 states with state laws or policies on sex and/or HIV education are shown (North Dakota and Wyoming are not represented).

**Figure 2:**
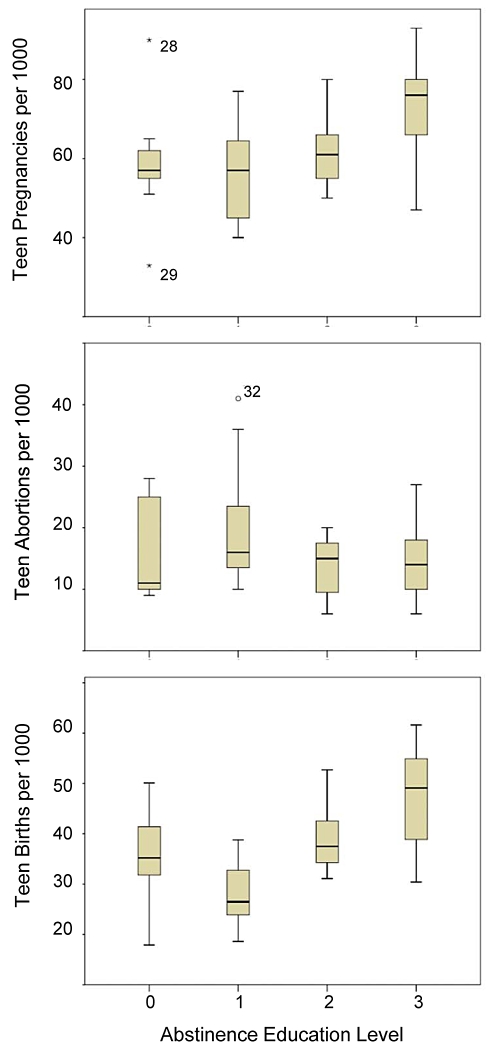
Mean teen pregnancy, abortion and birth rates by level of prescribed abstinence education. [Rates = numbers per 1000 girls 15–19 years old: shown are means ±2 SE]. Top panel: Teen pregnancies [outliers: #28 Nevada and #29 New Hampshire]; Middle panel: Teen abortions [outlier: #32 New York]; Bottom panel: Teen births. All outliers were included in the statistical analyses. A multivariate analysis of teen pregnancy and birth rates identified the level of abstinence education as a significant influence on teen pregnancy and birth rates across states.

Among the 48 states in this analysis (all U.S. states except North Dakota and Wyoming), 21 states stressed abstinence-only education in their 2005 state laws and/or policies (level 3), 7 states emphasized abstinence education (level 2), 11 states covered abstinence in the context of comprehensive sex education (level 1), and 9 states did not mention abstinence (level 0) in their state laws or policies (Figure 1). In 2005, level 0 states had an average (± standard error) teen pregnancy rate of 58.78 (±4.96), level 1 states averaged 56.36 (±3.94), level 2 states averaged 61.86 (±3.93), and level 3 states averaged 73.24 (±2.58) teen pregnancies per 1000 girls aged 14–19 (Table 3). The level of abstinence education (no provision, covered, promoted, stressed) was positively correlated with both teen pregnancy (Spearman's *rho* = 0.510, p = 0.001) and teen birth (*rho* = 0.605, p<0.001) rates (Table 4), indicating that abstinence education in the U.S. does not cause abstinence behavior. To the contrary, teens in states that prescribe more abstinence education are actually more likely to become pregnant (Figure 2). Abortion rates were not correlated with abstinence education level (*rho* = −0.136, p = 0.415). A multivariate analysis of teen pregnancy and birth rates identified the level of abstinence education as a significant influence on teen pregnancy and birth rates across states (pregnancies F = 5.620, p = 0.002; births F = 11.814, p<0.001). The significant pregnancy effect was caused by significantly lower pregnancy rates in level 0 (no abstinence provision) states compared to level 3 (abstinence stressed) states (p = 0.036), and level 1 (abstinence covered) states compared to level 3 states (p = 0.005); the significant birth effect was caused by significantly lower teen birth rates in level 0 states compared to level 3 (p = 0.006) states, and significantly lower teen birth rates in level 1 states compared to level 3 states (p<0.001).

**Table 3:** Teen pregnancy, abortion and birth rates (per 1000 girls aged 14–19) by level of abstinence education.

Descriptive Statistics by Abstinence Education Level						95% Confidence Interval			
Outcomes	Level	N	Median	Mean	Std. Error	Lower Bound	Upper Bound	Minimum	Maximum
Teen Pregnancies	0	9	57.0	58.78	4.966	47.43	70.23	33	90
	1	11	57.0	56.36	3.943	47.58	65.15	40	77
	2	7	61.0	61.86	3.931	52.24	71.47	50	80
	3	21	76.0	73.24	2.589	67.84	78.64	47	93
	Total	48	62.5	65.00	2.064	60.85	69.15	33	93
Teen Abortions	0	9	11.0	15.78	2.681	9.6	21.96	9	28
	1	11	16.0	20.27	3.069	13.43	27.11	10	41
	2	7	15.0	13.57	2.010	8.65	18.49	6	20
	3	21	12.0	14.86	1.306	12.13	17.58	6	27
	Total	48	15.00	16.08	1.096	13.88	18.29	6	41
Teen Births	0	9	35.2	34.82	3.316	22.8	41.5	18	50
	1	11	26.5	28.43	1.950	24.08	32.77	19	39
	2	7	40.0	39.29	2.765	32.52	46.05	31	53
	3	21	49.1	47.43	2.197	42.85	52.01	30	62
	Total	48	38.5	39.52	1.687	36.13	42.92	18	62

Based on 2005 data for all states except North Dakota and Wyoming, N = number of states.

**Table 4:** Socioeconomics and ethnic diversity as potential influences on teen pregnancy, abortion and birth rates in 48 states.

Correlation Coefficients		Teen Rates per 1000 girls (14–19)			Adjusted median household income	% Teens in population^1^		
		Pregnancies	Abortions	Births		White	Black	Hispanic
Abstinence Education level	Spearman's rho	0.507**	−0.083	0.562**	−0.349*	−0.382**	0.419**	0.030
	p (2-tailed)	**<0.001**	0.577	**<0.001**	**0.015**	**0.007**	**0.003**	0.839
Teen Pregnancies per 1000 girls	Spearman's rho		0.329*	0.806**	−0.383*	−0.807**	0.597**	0.341*
	p (2-tailed)		**0.022**	**<0.001**	**0.007**	**<0.001**	**<0.001**	**0.018**
Teen Abortions per 1000 girls	Spearman's rho			−0.221	−0.116	−0.564**	0.263	0.557**
	p (2-tailed)			0.131	0.432	**<0.001**	0.071	**<0.001**
Teen Births per 1000 girls	Spearman's rho				−0.296*	−0.482**	0.393**	0.036
	p (2-tailed)				**0.041**	**0.001**	**0.006**	0.806
Adjusted median income	Spearman's rho					0.298*	−0.238	0.089
	p (2-tailed)					**0.040**	0.103	0.547
% white teens in population	Spearman's rho						−0.566**	−0.532**
	p (2-tailed)						**<0.001**	**<0.001**
% black teens in population	Spearman's rho							−0.014
	p (2-tailed)							0.925

Significant correlations are marked in bold type (* significant at p<0.05, ** significant at p<0.01).

1 The % teen population variables are measures of the ethnic diversity of the states. Please note the teen pregnancy, abortion and birth data (per 1000) reflect the behavior of all teens in each state: they are not limited to the behavior within that particular ethnic teen population (see Table 5).

**Figure 3:**
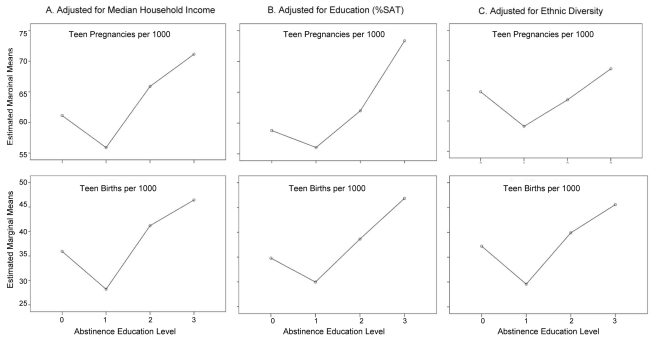
Trends in teen pregnancy and birth rates after accounting for socioeconomics, education and ethnic diversity. (A) The adjusted median household income significantly influenced teen pregnancy and birth rates, but the level of abstinence education still had a significant influence on teen pregnancy and birth rates after accounting for socioeconomic status. (B) Education had a significant influence on teen birth, but not on teen pregnancy rates. After accounting for the influence of teen education, the level of abstinence education still had a significant influence on both teen pregnancy and teen birth rates. (C) The proportion of white teens (but not black teens) in the population had a significant influence on teen pregnancy and teen birth rates. After accounting for this influence, the level of abstinence education still had a significant influence on teen pregnancy and birth rates.

Socio-economic status, educational attainment, and ethnic differences across states exhibited significant correlations with some variables in our model (Table 4). We examined the influence of each possible confounding factor on our analysis by including them as covariates in several multivariate analyses. However, after accounting for the effects of these covariates, the effect of abstinence education on teenage pregnancy and birth rates remained significant (Figure 3).

### Socio-economic status

There was a significant negative correlation between median household income (adjusted for cost of living) and level of abstinence education (*rho* = −0.349, p = 0.015; Table 4), indicating a socio-economic bias at the state level on state laws and regulations with regard to sex education. The adjusted median household income was negatively correlated with teen pregnancy (*rho* = −0.383, p = 0.007) and birth (*rho* = −0.296, p = 0.041) rates across states: pregnancy and birth rates tended to be higher in lower-income states. There was no correlation between household income and abortion rates (*rho* = −0.116, p = 0.432). When including the adjusted median household income as a covariate in a multivariate analysis (evaluated at $45,892), income significantly influenced teen pregnancy (F = 5.427, p = 0.025) but not birth (F = 2.216, p = 0.144) rates. After accounting for socioeconomic status, the level of abstinence education still had a significant effect on teen pregnancy (F = 4.103, p = 0.012) and birth rates (F = 10.480, p<0.001).

### Educational attainment

There was no significant correlation between statewide teen education (percentage of high school graduates that took the SAT in 2005/2006) and level of abstinence education (*rho* = −0.156, p = 0.291). Education was not correlated with teen pregnancy rates (*rho* = −0.014, p = 0.925), but it was positively correlated with teen abortion rates (*rho* = 0.662, p<0.001), and as a consequence, negatively correlated with teen birth rates (*rho* = −0.412, p = 0.004). There was no correlation between socio-economic status and teen educational attainment across states (*rho* = −0.048, p = 0.748), suggesting that these trends apply to both rich and poor states. When including education (% graduates taking the SAT) as a covariate in a multivariate analysis, education had a significant influence on teen birth (F = 8.308, p = 0.006), but not on teen pregnancy (F = 0.161, p = 0.690) rates, and after accounting for the influence of teen education (evaluated at 39.7% of graduates taking the SAT), the level of abstinence education still had a significant effect on both teen pregnancy (F = 5.527, p = 0.003) and teen birth rates (F = 10.772, p<0.001).

### Ethnic composition

For this analysis we focused on the three largest ethnic groups for which data are available: white, black, and Hispanic [12]. Teen pregnancy rates differ across these three ethnic groups. For the 48 states in this analysis, an ethnic breakdown (for all three ethnic groups) of teen pregnancy and abortion rates was available for 26 states, and of teen birth rates for 43 states. Across this reduced sample of states, 2005 teen pregnancy rates averaged 48.1 (±1.95) pregnancies per 1000 white teens, 103.7 (±5.38) pregnancies per 1000 black teens, and 141.6 (±8.55) pregnancies per 1000 Hispanic teens. Teen birth rates averaged 27.6 (±1.5) births per 1000 white teens, 59.2 (±2.58) births per 1000 black teens, and 96.1 (±5.39) births per 1000 Hispanic teens. Abstinence education levels were positively correlated with teen birth rates in all three ethnic groups (white: *rho* = 0.439, p = 0.002; black: *rho* = 0.328, p = 0.028; Hispanic: *rho* = 0.461, p = 0.001; Table 5).

**Table 5:** Ethnic breakdown of teen pregnancy, birth, and abortion rates and their relationship with abstinence education, educational attainment (SAT), adjusted income and teen diversity in the states.

Correlation Coefficients for ethnic diversity in states		Pregnancy rates (per 1000 girls)			Abortion rates (per 1000 girls)			Birth rates (per 1000 girls)		
		White	Black	Hispanic	White	Black	Hispanic	White	Black	Hispanic
Abstinence Education level	Spearman's rho	0.360	0.029	0.489*	0.024	−0.166	0.005	0.463**	0.332*	0.437**
	p (2-tailed)	0.071	0.890	**0.011**	0.909	0.417	0.980	**0.002**	**0.030**	**0.003**
Percent of graduates taking SAT	Spearman's rho	−0.134	0.053	0.104	0.723**	0.461*	0.613**	−0.450**	−0.504**	−0.258
	p (2-tailed)	0.514	0.796	0.614	**<0.001**	**0.018**	**0.001**	**0.002**	**0.001**	0.094
Adjusted median household income	Spearman's rho	−0.033	0.143	0.103	−0.348	−0.171	−0.240	−0.335*	0.106	0.099
	p (2-tailed)	0.873	0.486	0.617	0.081	−0.404	0.238	**0.028**	0.500	0.529
Proportion of white teens in population	Spearman's rho	−0.307	0.054	−0.318	−0.376	−0.015	−0.256	−0.017	0.162	0.064
	p (2-tailed)	0.127	0.794	0.114	0.058	0.944	0.206	0.916	0.298	0.685
Proportion of black teens in population	Spearman's rho	0.550**	0.539**	0.393*	0.113	0.086	0.031	0.282	0.420**	0.215
	p (2-tailed)	**0.004**	**0.004**	**0.047**	0.584	0.675	0.880	0.067	**0.005**	0.166
Proportion of hispanic teens in population	Spearman's rho	−0.366	−0.226	0.071	0.093	0.108	0.262	−0.434**	−0.347*	−0.140
	p (2-tailed)	0.066	0.267	0.730	0.652	0.600	0.196	**0.004**	**0.023**	0.370

Sample sizes for the analysis of ethnic breakdown (for all three ethnic groups) of teen pregnancy and abortion (N = 26 states) and birth rates (N = 43 states) are limited. Significant correlations are marked in bold type (* significant at p<0.05, ** significant at p<0.01).

Across all 48 states, abstinence education levels were significantly correlated with the proportions of white and black teens in the state populations (Table 4). In general, states with higher proportions of white teens tended to emphasize abstinence less (*rho* = −0.382, p = 0.007), and states with higher proportions of black teens tended to emphasize abstinence more (*rho* = 0.419, p = 0.003). When we included the proportion of white and black teens in the state populations as covariates in a multivariate analysis (evaluated at proportion white: 0.704 and proportion black: 0.138), only the proportion of white teens had a significant effect on teen pregnancy (F = 42.206, p<0.001) and teen birth rates (F = 5.894, p = 0.020). After accounting for this influence, the level of abstinence education still had a significant effect on teen pregnancy (F = 2.839, p = 0.049) and teen birth rates (N = 43 states: F = 7.782, p<0.001; Figure 3).

### Medicaid waivers

**Figure 4:**
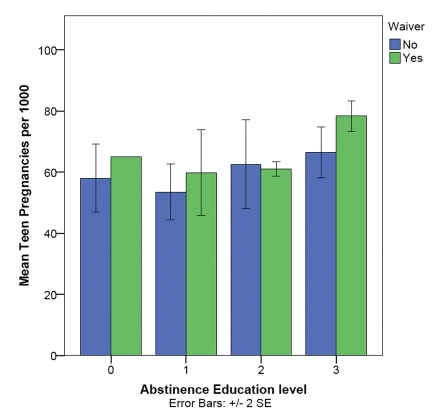
Teen pregnancy rates, abstinence education levels and Medicaid waivers to access family planning services. Access to waivers does not explain the difference in teen pregnancy rates (shown are means and ±2 SE) in states with a different emphasis on abstinence.

If Medicaid waivers contribute to the positive correlation between abstinence education and teen pregnancy at the state level, then states with waivers should have different teen pregnancy and birth rates than states without waivers. This was not the case. States with waivers (N = 17) were represented across all four abstinence education levels (Figure 4) and did not differ significantly in teen pregnancy rates from states without waivers (N = 21, Mann Whitney U = 237, p = 0.086), suggesting no significant effect of waivers (at the state level) on the correlation between abstinence levels and teen pregnancy rates. A recent study [14] found the same level of (non-)significance (0.05<p<0.1) for the effect of waivers on teen birth rates, but reported it as significant.

## Discussion

This study used a correlational approach to assess whether abstinence-only education is effective in reducing U.S. teen pregnancy rates. Correlation can be due to causation, but it can also be due to other underlying factors, which need to be examined. Several factors besides abstinence education are correlated with teen pregnancy rates. In agreement with previous studies, our analysis showed that adjusted median household income and proportion of white teens in the teen population both had a significant influence on teen pregnancy rates. Richer states tend to have a higher proportion of white teens in their teen populations, tend to emphasize abstinence less, and tend to have lower teen pregnancy and birth rates than poorer states. A recent study [25] found that higher teen birth rates in poorer states were also correlated with a higher degree of religiosity (and a lower abortion rate) at the state level. Medicaid waivers have previously been shown to reduce teen pregnancy rates [13], but our analysis shows that they do not explain our main result, the positive correlation between abstinence education level and teen pregnancy rates.

After accounting for other factors, the national data show that the incidence of teenage pregnancies and births remain positively correlated with the degree of abstinence education across states: The more strongly abstinence is emphasized in state laws and policies, the higher the average teenage pregnancy and birth rate. States that taught comprehensive sex and/or HIV education and covered abstinence along with contraception and condom use (level 1 sex education; also referred to as “abstinence-plus” [26], tended to have the lowest teen pregnancy rates, while states with abstinence-only sex education laws that stress abstinence until marriage (level 3) were significantly less successful in preventing teen pregnancies. Level 0 states present an interesting sample with a wide range of education policies and variable teen pregnancy and birth data [17]–[19]. For example, several of the level 0 states (as of 2007) did not mandate sex education, but required HIV education only (e.g. CT, WV) [19]. Only three of the level 0 states (IA, NH and NV) mandated both sex education and HIV education, but one of them (NV) did not require that teens learn about condoms and contraception. This state (NV) has the highest teen pregnancy and birth rates in that group (Figure 1). Nevada is also one of only five states (with MD in level 0, CO in level 2, and AZ and UT in level 3) that required parental consent for sex education in public schools instead of an opt-out requirement that is present in all the other states [16], [19].

The effectiveness of Level 1 (comprehensive) sex education in our nation-wide analysis is supported by Kirby's meta-analysis of individual sex education programs [8], Underwood et al. 's analysis of HIV prevention programs [27], and a recent review by the CDC taskforce on community preventive services [28]. All these studies suggest that comprehensive sex or HIV education that includes the discussion of abstinence as a recommended behavior, and also discusses contraception and protection methods, works best in reducing teen pregnancy and sexually transmitted diseases.

### Individual research studies

Despite large differences between individual research studies that evaluate specific sex education programs (e.g. sample size, approaches to sex education studied, selection of participants, choice of control groups, types of data, control for cross-talk between students outside of class, etc.), several case studies show that abstinence-only education rarely has a positive effect on teen sexual behavior [6], [8], [29]. One of the few exceptions is the recent study by Jemmott et al. [30] on black middle school students in low-income urban schools: after receiving 8 hours of abstinence education as 12 year olds, significantly more students (64/95) reported to be abstinent after 24 months when compared to (control) students who received 8 hours of health education (without any form of sex education: 47/88; Fishers exact test, p = 0.037), or students who received 8 hours of safe-sex education (without an abstinence component: 41/85, Fishers exact test, p = 0.007). However, there was no significant difference in abstinence behavior between students who had received abstinence education (64/95) and students who received 8 hours of comprehensive sex education (combining sex education with abstinence education: 57/97; Fishers exact test, p = 0.138). These two groups also did not differ in rates of reported unprotected sex (8/122 versus 8/115) or use of condoms (25/33 versus 29/37) in the previous 3 months. The abstinence-only intervention in that study was unique in that it increased knowledge about HIV/STD, emphasized the delay of sexual activity, but not necessarily until marriage, did not put sex into a negative light or use a moralistic tone, included no inaccurate information, corrected incorrect views, and did not disparage the use of condoms [30]. As a result, as pointed out by the authors, this successful version of abstinence education would not have met the criteria for federal abstinence-only funding [30]. While promoting an alternative and more effective form of abstinence education, these results also support Kirby's findings [8] and the data in the present study that comprehensive sex education that includes an abstinence (delay) component (level 1), is the most effective form of sex education, especially when using teen pregnancy rates as a measurable outcome.

Individual research studies also show that teaching about contraception is generally not associated with increased risk of adolescent sexual activity or sexually transmitted diseases (STDs) [8] as suggested by abstinence-only advocates, and adolescents who received comprehensive sex or HIV education had a lower risk of pregnancy and HIV/STD infection than adolescents who received strict abstinence-only or no sex education at all in the U.S. and in other high-income countries [27], [31].

### Abstinence-only education: public opinion and associated costs

Despite the data showing that abstinence-only education is ineffective, it may be argued that the prescribed form of sex education represents the underlying social values of families and communities in each state, and changing to a more comprehensive sex education curriculum will meet with strong opposition. However, there is strong public support for comprehensive sex education [32]. Approximately 82% of a randomly selected nationally representative sample of U.S. adults aged 18 to 83 years (N = 1096) supported comprehensive programs that teach students about both abstinence and other methods of preventing pregnancy and sexually transmitted diseases. In contrast, abstinence-only education programs, received the lowest levels of support (36%) and the highest level of opposition (about 50%).

In addition to the federal and state funds spent on abstinence-only (level 3) education, there are other costs associated with the outcomes of failed sex education and family planning. When deciding state policies on sex education, State legislators should consider these additional costs. For example, based on estimates by the *National Campaign To Prevent Teen and Unplanned Pregnancy* [33], teen child bearing (compared to first birth at 20 years or older) in the U.S. cost taxpayers (in direct and indirect costs) more than $9.1 billion in 2004.

Our data show that education (% of high school graduates taking the SAT) was not correlated with teen pregnancy rates, but it was positively correlated with teen abortion rates and negatively correlated with teen birth rates. These data can be interpreted in two ways: (1) pregnant teens who give birth are less likely to finish high school and go on to college (i.e. pregnancy affects education). This is supported by a recent report [34] that showed that teen mothers are more likely to drop out of school: 51% of teen mothers earned their high school diploma by age 22, compared to 89% of women who had not given birth as teens. (2) teens who are motivated to go to college are not necessarily less likely to get pregnant, but more likely to abort their pregnancies (i.e. educational goal affects the decision of whether to carry a pregnancy to term).

As pointed out by the Society for Adolescent Medicine, the abstinence-only approach (as stressed by level 3 state laws and policies and funded by the federal abstinence-only programs) is characterized by the withholding of information and is ethically flawed [7]. Abstinence-only programs tend to promote abstinence behavior through emotion, such as romantic notions of marriage, moralizing, fear of STDs, and by spreading scientifically incorrect information [7], [20], [35]. For example a Congressional committee report found evidence of major errors and distortions of public health information in common abstinence-only curricula [36]. As a result, these programs may actually be promoting irresponsible, high-risk teenage behavior by keeping teens uneducated with regard to reproductive knowledge and sound decision-making instead of giving them the tools to make educated decisions regarding their reproductive health [37]. The effect of presenting inadequate or incorrect information to teenagers regarding sex and pregnancy and STD protection is long-lasting as uneducated teens grow into uneducated adults: almost half of all pregnancies in the U.S. were unplanned in 2001 [38]. Of these three million unplanned pregnancies, ∼1.4 million resulted in live births, ∼1.3 million ended in abortion, and over 400,000 ended in a miscarriage [36], [37] at a financial cost (direct medical costs only) of ∼$5 billion in 2002 [39].

The U.S. teen pregnancy rate is substantially higher than seen in other developed countries (Table 1) despite similar cultural and socioeconomic patterns in teen pregnancy rates [40]. The difference is not due to the onset of sexual activity [1]. Instead, the main factor seems to be sex education, especially with regard to contraception and prevention of STDs [41]. Sex education in Europe is based on the WHO definition of sexuality as a lifelong process, aiming to create self-determined and responsible attitudes and behavior with regard to sexuality, contraception, relationships and life strategies and planning [42]. In general, there is greater and easier access to sexual health information and services for all people (including teens) in Europe, which is facilitated by a societal openness and comfort in dealing with sexuality [40], by pragmatic governmental policies [43], [44] and less influence by special interest groups.

### Future Directions

While states with comprehensive sex education have lower teen pregnancy rates, even in these states rates are much higher than seen in Europe [1]. This is likely influenced by the fact that U.S. state laws and policies generally do not require that sex and STD education is taught in all schools, but only provide guidelines if local school boards decide to teach it [19]. For example, as of August 1, 2011, only 20 states mandated sex education, and 32 states mandated HIV education in their schools [45]. In addition, even states with comprehensive sex education laws or policies (level 1) received federal funding for individual abstinence-only education programs in 2005: total federal funds [19] averaged ∼$14 per teen in level 1 states compared to ∼$21 per teen in level 2 and 3 states [12]. An important first step towards lowering the high teen pregnancy rates would be states requiring that comprehensive sex education (with abstinence as a desired behavior) is taught in all public schools. Another important step would involve specialized teacher training. Presently the sex education and STD/HIV curricula are often taught by faculty with little training in this area [46]. As a further modification, “sex education” could be split into a coordinated social studies component (ethics, behavior and decision-making, including planning for the future) and a science component (human reproductive biology and biology of STDs, including pregnancy and STD prevention), each taught by trained teachers in their respective field.

**Figure 5:**
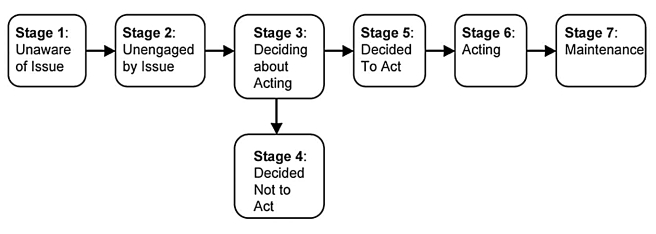
The Precaution-Adoption-Process Model. This model offers a basis for communication and discussions between educators, scientists, sex education researchers, and health professionals, and could serve as a reference for measuring progress in sex education. In addition, it could be used as a communication tool between sex education teachers and their students [48].

As parents, educators or policy makers it should be our goals that (1) teens can make educated reproductive and sexual health decisions, that (2) teen pregnancy and STD rates are reduced to the rates of other developed nations, and that (3) these trends are maintained through the teenage years into adulthood. One possibility for achieving these goals is a close alignment and integration of sex education with the National Science Standards for U.S. middle and high schools [47]. In addition, the *Precaution Adoption Process Model* (Figure 5) advocated by the National Institutes of Health [48] offers a good basis for communication and discussions between scientists, educators, and sex education researchers, and could serve as a reference for measuring progress in sex education (in alignment with the new evidence-based Teen Pregnancy Prevention Initiative). In addition, it could be used as a communication tool between sex education teachers and their students. It should be our specific goal to move American teens from Stages 1 or 2 (unaware or unengaged in the issues of pregnancy and STD prevention) to Stages 3–7 (informed decision-making) by providing them with knowledge, understanding, and sound decision-making skills (Figure 5). For example, a recent study [49] attributes 52% of all unintended pregnancies (teenagers and adults) in the U.S. to non-use of contraception, 43% to inconsistent or incorrect use, and only 5% to method failure.

Our analysis adds to the overwhelming evidence indicating that abstinence-only education does not reduce teen pregnancy rates. Advocates for continued abstinence-only education need to ask themselves: If teens don't learn about human reproduction, including safe sexual health practices to prevent unintended pregnancies and STDs, and how to plan their reproductive adult life in school, then when should they learn it, and from whom?
